# Fluoride Uptake and Surface Characteristics of Ion-Releasing Restoratives After Brushing with Fluoride Toothpastes

**DOI:** 10.3390/ma18092152

**Published:** 2025-05-07

**Authors:** Llubitza Slaviza Banic Vidal, Ivan Šalinović, Nikolina Nika Veček, Anja Ivica, Ivana Miletić, Silvana Jukić Krmek

**Affiliations:** 1 School of Dental Medicine, University of Zagreb, Gundulićeva 5, 10000 Zagreb, Croatia; slallu@hotmail.com; 2 Department for Endodontics and Restorative Dentistry, School of Dental Medicine, University of Zagreb, Gundulićeva 5, 10000 Zagreb, Croatia; aivica@sfzg.unizg.hr (A.I.); miletic@sfzg.unizg.hr (I.M.); jukic@sfzg.unizg.hr (S.J.K.); 3 School of Medicine, University of Split, Šoltanska 2A, 21000 Split, Croatia; veceknika@gmail.com

**Keywords:** ion-releasing materials, toothpaste, brushing protocol, fluoride uptake, surface roughness, microhardness

## Abstract

This study aimed to evaluate the influence of different fluoride-containing toothpastes on fluoride uptake, surface roughness, and microhardness of six ion-releasing restorative dental materials, including glass hybrids (EQUIA Forte HT with and without coating), glass ionomer cements (Fuji IX), resin-modified GICs (Fuji II LC), alkasites (Cention Forte), and ion-releasing composites (Luminos UN and Activa). Specimens were prepared and subjected to a four-day brushing protocol using six toothpastes with varying fluoride formulations (NaF, SnF2, SMFP) and concentrations. Fluoride uptake was assessed by measuring fluoride release using an ion-selective electrode, while surface roughness and microhardness were assessed before and after brushing. Results revealed significant variations in fluoride uptake, with Fuji IX and EQUIA Forte HT showing the highest release, particularly when brushed with NaF-based toothpastes (Duraphat 5000 and 2800). Surface roughness increased post-brushing, with the greatest changes observed in Activa, while microhardness decreased across most materials, except for coated EQUIA Forte HT, which exhibited improved compactness. Resin-based composites, such as Luminos UN and Activa, demonstrated lower fluoride uptake and minimal changes in microhardness compared to GICs. The findings underscore the importance of material composition and toothpaste formulation in influencing fluoride dynamics, surface properties, and mechanical performance of restorative materials.

## 1. Introduction

The effects of fluoride use on dental caries prevention are among the most extensively studied topics in dentistry. Although significant epidemiological evidence supports its effectiveness in various forms, such as toothpaste, mouth rinses, and drinking water, the exact mechanism by which it works is still not fully understood [1] but includes the formation of less acid degradation-prone fluorapatite and bacterial metabolism modification [2,3]. Capitalizing on fluoride’s therapeutic benefits, manufacturers of restorative dental materials have developed a variety of products designed to enhance their levels in the oral cavity [4]. Previously being present mostly in oral hygiene products and for systemic intake, fluorides appeared in restorative products with the introduction of glass ionomer cements [GICs], which are still commonly used. Their low mechanical resilience resulted in different modifications over the years, which improved their mechanical properties [5,6]. Therefore, materials like resin-modified glass ionomer cement, glass hybrids, and alkasites have emerged on the market. Resin-based composites, a gold standard material for direct restorations, have also been modified with the addition of fluorides in different hybrid versions [7].

While the release of fluorides from the restorative materials is crucial for them to combat caries formation and progression [8], these materials are meant to be efficient to a certain degree for as long as they are present in the mouth, meaning that they should also be able to absorb the fluoride ions from their surroundings [9,10]. Fluoride uptake, similar to fluoride release, is a dynamic process, best described in glass ionomer cements, where fluoride ions bind chemically to the cement and can be released, depending on the circumstances in the oral cavity [11,12]. The fluoride uptake of other restorative materials is still not investigated enough.

The prevailing technique for delivering fluoride ions into the oral cavity and preventing dental caries is the use of fluoride-containing toothpaste [13]. Toothpastes are classified as a semi-solid substance formulated to eliminate natural deposits from the teeth and adjacent tissues and are intended for use in combination with a toothbrush [14]. In 2019, the World Health Organization (WHO) issued a statement with the recommended amount of fluorides of 1000 to 1500 ppm as a standard in toothpastes, with variants of 2800–5000 ppm being available for high-risk patients [15]. Previous research suggests that fluoride-containing toothpastes are around 25% more effective than non-fluoridated toothpastes in caries reduction [16]. The three primary sources of fluoride used globally are stannous fluoride (SnF_2_), sodium fluoride (NaF), and sodium monofluorophosphate (Na_2_PFO_3_ or SMFP). Stannous fluoride is effective in preventing dental caries and possesses antibacterial properties. Its ability to adhere to tooth surfaces enhances its protective role and supports the remineralization of enamel, while also alleviating dental hypersensitivity [17]. NaF is widely used in dental care products for its potent caries prevention capabilities. It facilitates the remineralization of enamel and increases the acid resistance of teeth, making it a key component in toothpaste and professional treatments [18]. SMFP releases fluoride during hydrolysis, contributing to enamel remineralization. Its stability and compatibility with other ingredients make it a favorable option for incorporation into toothpaste formulations, ensuring effective fluoride delivery [19]. While toothpastes can boost caries prevention, the common presence of silica and other hard particles can lead to erosive wear of both dental tissues and restorative materials [20]. It is still under investigation whether the effect of toothpastes on the material’s surface can affect its ion exchange properties.

Many studies have successfully measured fluoride uptake using an ion-selective electrode, which detects the amount of fluoride released and tracks changes in concentration over time [21,22]. Most of these studies involved soaking the samples in toothpaste slurries without incorporating brushing. However, brushing action can affect the surface and mechanical properties of the materials, potentially altering the interaction between the toothpaste and the material. This, in turn, may influence the ion-exchange properties.

The introduction of new ion-releasing materials to the market is not followed by the appropriate number of studies. As the fluoride exchange between the material and the surrounding environment is considered of extreme importance for many of their effects, the aim of this study was to determine the influence that different types of toothpastes have on the fluoride uptake, microhardness, and surface roughness in ion-releasing restorative materials. The null hypothesis was that all the examined materials would be affected in a similar way, regardless of the type and concentration of fluoride in the toothpastes.

## 2. Materials and Methods

Six fluoride-containing restorative dental materials were included in this study. Their compositions, as provided by the manufacturers, are available in Table 1.

Six different toothpastes were used during the brushing period. They are listed in Table 2.

### 2.1. Specimen Preparation

Teflon molds were used to prepare cylindrical disc-shaped specimens of each material (8 mm in diameter and 2 mm wide). For each material, ten test samples were prepared (n = 10), along with the same number of samples for the control group that were not exposed to the brushing protocol. Most materials were in encapsulated form and mixed using a Silver Mix capsule mixer (GC Corporation, Tokyo, Japan), except Luminos UN and Activa, which did not require any preparation. To minimize air entrapment, polyester strips and glass plates were used to gently compress the material in the molds after the application. Light-curable materials (Luminos UN, Activa, Fuji II LC, Cention Forte) were polymerized for 40 s using the light cure unit Woodpecker LED-C (Guilin Tucano Medical Apparatus and Instruments Limited Company, Guilin, China) with curing light output: 850 W/cm^2^ and wavelength: 420–480 nm. Self-curing materials were allowed to chemically set for one hour before demolding. For EQUIA Forte HT, the coated samples received additional surface treatment: the coating was applied with a brush provided by the manufacturer, air-dried for 5 s, and light-cured for 10 s using the same unit described above.

All samples were left to set for one hour at 37 °C and individually placed in polyethylene vials (12 mm wide and 38 mm high) (Laboroprema, Zagreb, Croatia) containing 5 mL of deionized water. They were aged for 5 weeks at 37 °C in a cooled incubator (ES 120, NÜVE, Ankara, Turkey) to ensure material exhaustion prior to testing.

### 2.2. Brushing Protocol

**Figure 1:**
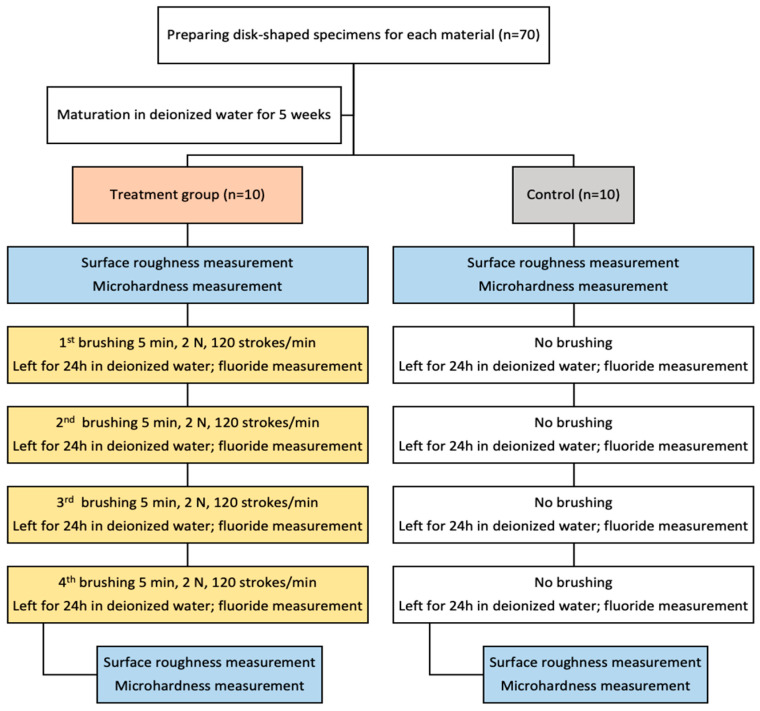
Flowchart of the study.

A custom-built device for brushing simulation was used, manufactured as a result of interdisciplinary teamwork. Ten Colgate Extra Clean Medium brush heads (Colgate-Palmolive Europe, Therwil, Switzerland) were used, each featuring round-ended nylon bristles (12 mm length, arranged in 35 tufts). The brush heads were securely fastened with screws and connected to a central drive system, ensuring consistent movement across all test specimens. During testing, the toothbrush heads were positioned parallel to the sample surfaces to standardize contact and abrasion conditions. As a medium for brushing, a toothpaste slurry was prepared and replaced after each cycle, containing 50 g of tested toothpaste, 0.624 g tragacanth gum, E413, 24.69 g of deionized water, and 24.69 g of glycerin (Glaconcheme GmbH, Merseburg, Germany), with a magnetic mixer (Intllab, China). The slurry was based on ISO 11609 standards [23]. To mimic daily cumulative brushing, the top side of each specimen was brushed for a total of 5 min. The cleaning force was set to 2 N, using an 8 mm longitudinal and 8 mm transverse motion at a frequency of 120 strokes per minute. Specimens were randomly assigned to either the brushing or control groups. Ten specimens from the same group were simultaneously exposed to abrasion in the tooth-brushing machine. After each brushing, specimens were thoroughly rinsed with deionized water and stored in a vial (Laboroprema, Zagreb, Croatia) containing 5 mL of deionized water at 37 °C under 100% humidity. Fluoride concentration was determined 24 h after brushing. Brush heads were cleaned with deionized water and the brushing procedure was repeated for four days, as explained in Figure 1. The same toothbrushes were used throughout the study.

### 2.3. Fluoride Uptake Measurement

Fluoride uptake was quantified using an ion-selective electrode (ISE) to detect fluoride release 24 h after each brushing cycle, conducted over four consecutive days. For each measurement, the discs were removed from water, gently blotted dry using lint-free paper to eliminate surface moisture without damaging the sample surface, and subsequently transferred into individual vials containing 5 mL of freshly prepared deionized water for fluoride elution. Fluoride concentrations were measured daily using an ion-selective electrode F800 DIN (Xylem Analytics, Weilheim, Germany) coupled with an ion analyzer inoLab Multi 9630 DS (Xylem Analytics, Weilheim, Germany). Prior to analysis, the ISE was prepared with WTW outer chamber filling solution (ELY/BR/503), as recommended by the manufacturer, and calibrated using fluoride standard solutions (10, 20, and 50 mg/L) to generate a standard calibration curve, ensuring accurate quantification across the expected concentration range. After passing quality control checks, 5 mL of TISAB II solution (Total Ionic Strength Adjustment Buffer, Merck KGaA, Darmstadt, Germany) was added to each sample to maintain constant ionic strength, pH stability, and to eliminate potential interferences from complexing ions. Measurements were performed in triplicate, and the mean fluoride concentration was recorded in mg/L (ppm F^−^).

### 2.4. Surface Roughness Determination

Surface roughness was measured using a portable roughness tester (Surftest SJ-210; Mitutoyo, Houston, TX, USA) with a diamond tip of 5 μm radius and the following settings: stylus speed of 0.1 mm/s, stylus force of 4 mN, cut-off length of 0.25 mm, and sampling length of 0.8 mm. Initial measurements were taken immediately after the samples were removed from their vials following 5 weeks of storage and before brushing. Final measurements were recorded after completing all four brushing cycles. Each specimen underwent three measurements at distinct points within a 5 mm diameter from the center, and the mean Ra value was calculated as the representative surface roughness.

### 2.5. Microhardness Measurement

Microhardness testing was performed using a Qness Q10 M Microhardness Tester (ATM Qness GmbH, Golling an der Salzach, Austria). Measurements were taken immediately after the samples underwent surface roughness measuring and prior to brushing. The Vickers method (HV0.1) was applied, utilizing a 100 g load for 10 s. Three indentations were made per sample, and their arithmetic mean was used as the representative value. To maintain measurement accuracy, the spacing between indentation marks was at least three times their diameter. After completing all four brushing cycles, microhardness testing was repeated following the same protocol.

### 2.6. Statistical Analysis

The data were analyzed using repeated measures analysis of variance with 4 related variables (fluoride levels on days 1, 2, 3, and 4) across 42 independent groups (6 toothpastes and 7 dental materials). The test/retest reliability of measurement was tested using the Pearson correlation coefficient. The differences between individual materials and toothpastes were tested using a post hoc Least Significant Difference (LSD) test. All analyses were performed using IBM SPSS software, v. 21. (IBM, Armonk, NY, USA). The statistical significance was set at *p* < 0.05.

## 3. Results

### 3.1. Fluoride Uptake

**Figure 2:**
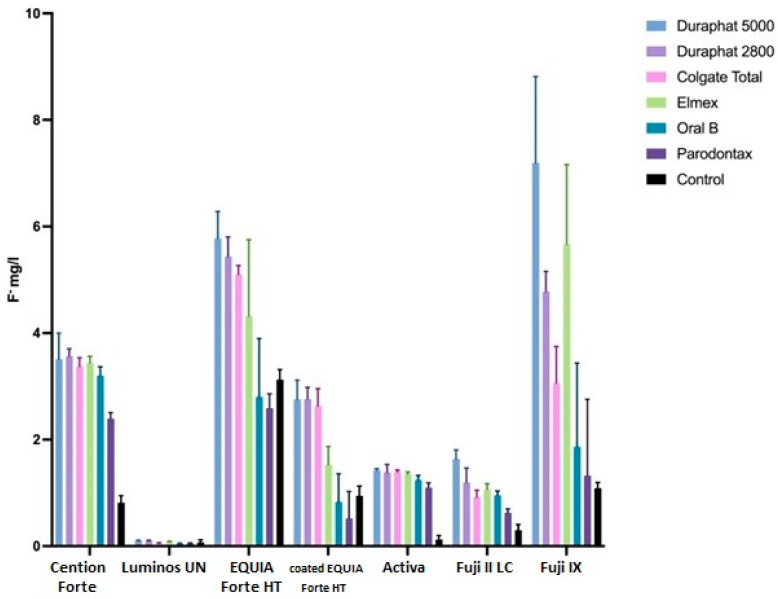
Average fluoride release (mg/L), reciprocal with fluoride uptake, from tested materials, as influenced by different toothpaste during brushing protocol. Error bars represent standard deviation.

**Figure 3:**
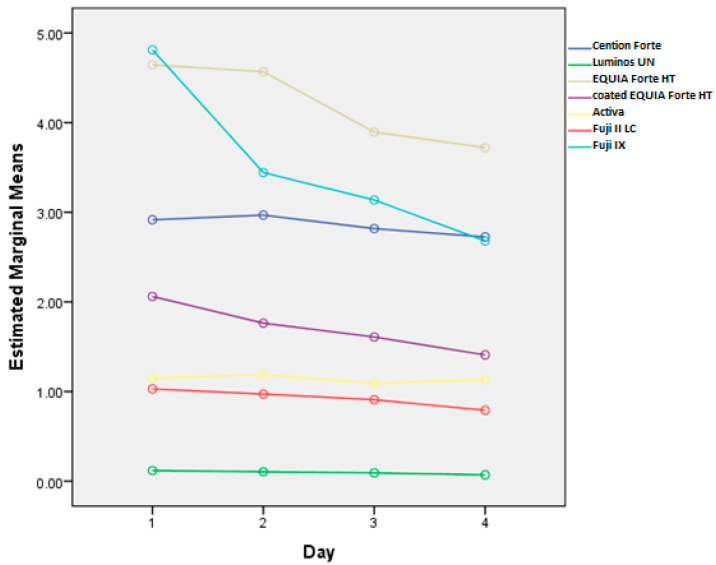
Overall fluoride release (mg/L) by day for each tested material. The data illustrate the variation in fluoride release across different materials over four days.

**Figure 4:**
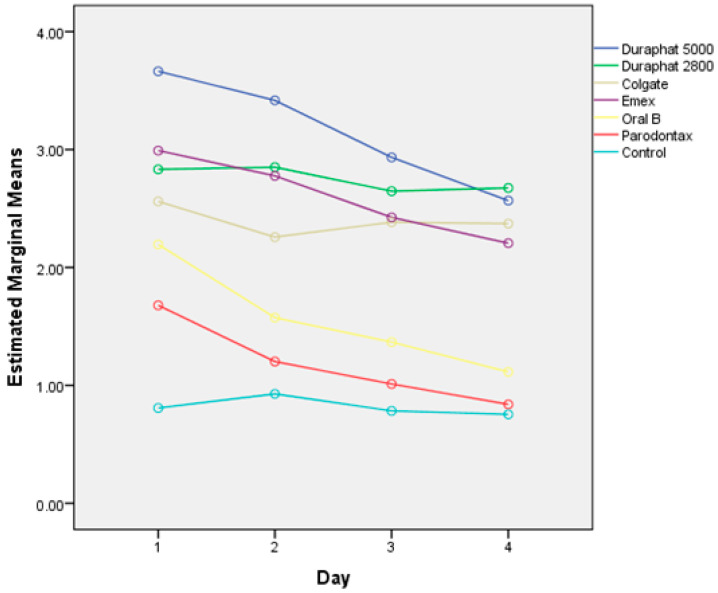
Overall fluoride release (mg/L), corresponding to uptake, after brushing with different toothpaste samples and control samples without toothpaste.

Cumulative fluoride release rates, corresponding to fluoride uptake, from tested materials after the brushing protocol are shown in Figure 2, while the daily changes in release rates are shown in Figure 3. The effects of different toothpastes on fluoride release in general are shown in Figure 4.

There are statistically significant differences between the subsequent measurements overall (*p* < 0.0001). These differences are significantly dependent on toothpaste (*p* < 0.001) and material (*p* < 0.001). Also, the effect of toothpaste is different for different materials (*p* < 0.001).

Overall, the cumulative fluoride release (all materials together) is the highest for Duraphat 5000 toothpaste, significantly higher than other toothpastes or control, followed by Duraphat 2800 and Elmex (not significantly different from each other), followed by Colgate Total, then Oral B and Parodontax. The control had the lowest values (all differences statistically significant at *p* < 0.001. When compared by material, in all materials the differences between toothpastes are statistically significant (*p* = 0.006 for Lumia, *p* < 0.001 for other materials), but comparing individual toothpastes within each specific material shows variations in the sense that the differences are smaller in some materials (and sometimes not significant), but overall picture remains similar, with one notable exception: Elmex has significantly higher fluoride release than Duraphant 2800 and Colgate Total in Fuji IX samples (*p* < 0.001), but significantly lower in coated EQUIA Forte HT samples (*p* < 0.001).

### 3.2. Surface Roughness

**Figure 5:**
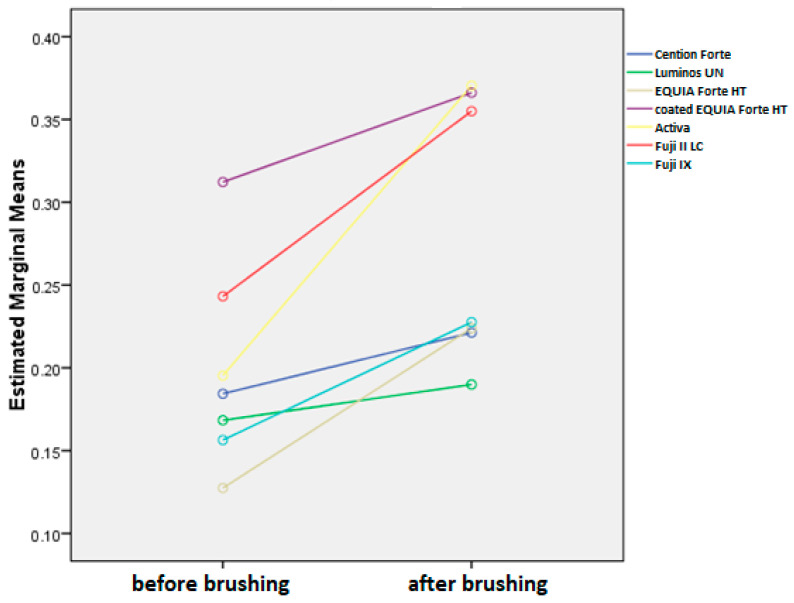
Mean surface roughness values (Ra) before and after brushing protocol for all toothpastes combined.

Brushing significantly increases surface roughness (*p* < 0.001), but this effect is significantly different across materials (*p* < 0.001) and toothpastes (0.001) and even their interactions (*p* < 0.001), as shown in Figure 5. It must be noted that surface roughness is different in different materials, and these differences are present after brushing as well.

**Figure 6:**
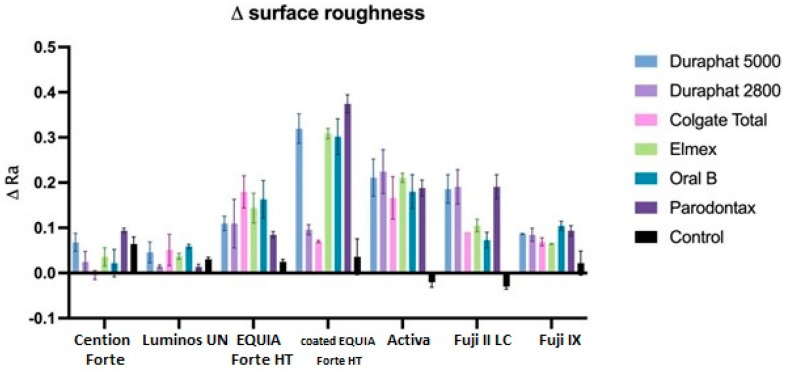
Surface roughness (Ra values) changes between initial and final values. All data are represented as mean values.

Overall, the average reduction of surface roughness across all materials and toothpastes is 0.08+/−0.08. When comparing this increase by material, we can see that the average change is the highest in Activa, followed by Fuji II LC, EQUIA Forte HT, Fuji IX, coated EQUIA Forte HT, Cention Forte, and Luminos UN (Figure 6). The differences between Activa and all other materials are significant (*p* < 0.001), while other materials are significantly different from materials two places further down, but not the next one (EQUIA Forte HT is not significantly different from Fuji II or Fuji IX with *p* > 0.05 but is different from all other materials at *p* < 0.001. Likewise, Fuji IX is not different from EQUIA Forte HT or coated EQUIA Forte HT but is significantly different at *p* < 0.001 from all other materials).

When comparing this reduction by toothpaste, we can see that all toothpastes are significantly different than control (*p* < 0.001) and not different from each other, except for Parodontax, which is significantly different than Colgate Total (*p* < 0.05). Regarding the combinations of toothpaste and material, the highest surface roughness increase reduction is seen for Duraphate 2800, Duraphate 5000, Elmex, and Parodontax toothpastes on Activa material (the difference is statistically significant between these combinations and EQUIA Forte HT, coated EQUIA Forte HT, and Luminos UN compared to the control group).

### 3.3. Microhardness Measurement Results

**Figure 7:**
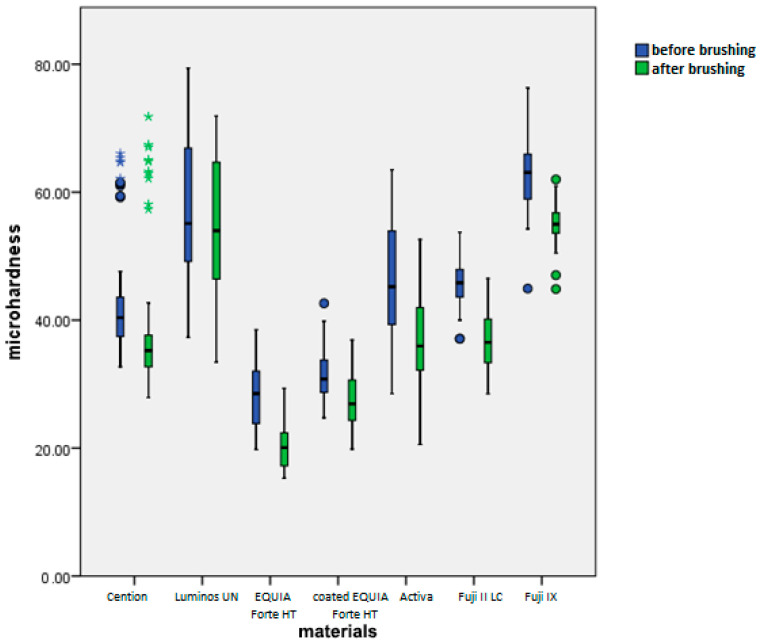
Mean microhardness values (HV 0.1) before and after brushing by material.

Brushing significantly reduces microhardness (*p* < 0.001), but this effect is significantly different across materials (*p* < 0.001) and toothpastes (0.001) and even their interactions (*p* < 0.001). It must be noted that microhardness is different in different materials and these differences are present after brushing as well (Figure 7).

**Figure 8:**
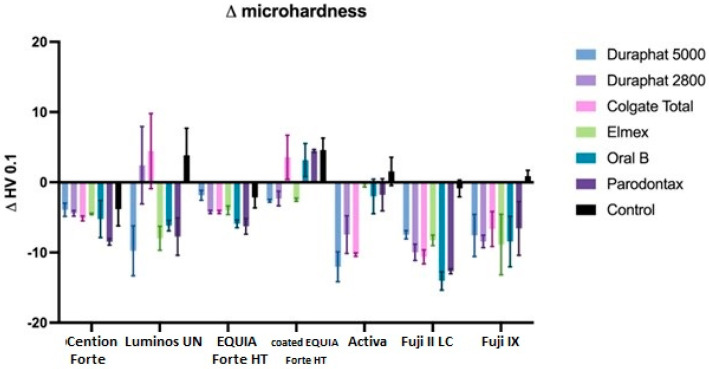
Microhardness changes between initial and final values. All data are represented as mean ± standard deviation.

Overall, average reduction of microhardness across all materials and toothpastes is 6.56+/−5.07 (Figure 8). When comparing this reduction by material, we can see that the average change is the highest in Activa and Fuji II LC. The next group is Fuji II LC, EQUIA Forte HT, and Fuji IX. The last homogenous group includes coated EQUIA Forte HT, Cention Forte, and Luminos UN. Differences among materials within this group were not statistically significant (*p* > 0.05), whereas differences between this group and other groups were statistically significant (*p* < 0.01).

When comparing this reduction by toothpaste, we can see that all toothpastes are significantly different than the control (*p* < 0.001 for Elmex, Oral B, and Parodontax, *p* < 0.05 for Duraphat 5000) and not different from each other, except for Duraphat 2800, which is not significantly different than the control (*p* = 0.05), but also not different than any other toothpaste (*p* > 0.05).

There are statistically significant differences between materials in obtained microhardness values after brushing, with the highest microhardness exhibited by Fuji IX and Luminos UN, followed by Cention Forte, Fuji II LC, and Activa, followed by coated EQUIA Forte HT, and lowest microhardness for EQUIA Forte HT (all *p* values < 0.001) for materials in different groups.

When comparing toothpastes, the highest microhardness is found in the control group (significantly different than all toothpastes, *p* < 0.01). Among toothpastes, the only significant difference is found between Duraphat 2800 and Colgate Total, which are significantly higher in final microhardness than Duraphat 5000 (*p* < 0.05).

## 4. Discussion

The results of this study demonstrate significant variations in fluoride uptake, release, surface roughness, and microhardness depending on the measurement points and toothpaste used. Consequently, the initial hypothesis has been rejected. Fluoride uptake was assessed using a well-established method that measures fluoride release before and after immersion, as validated by multiple studies [24,25,26,27]. Thus, it is used in the present study. A key novelty of this study is the inclusion of a brushing protocol before immersion. The reliability of the fluoride release measurements was high, with Pearson correlation coefficients ranging from 0.9907 to 0.9993. This confirms high measurement consistency and reliability, with minimal error variance. Furthermore, similar to the findings of the current study, materials with higher fluoride release are also expected to release more ions after ion uptake [28]. This protocol aimed to replicate certain parameters of actual toothbrushing by using a standard type of toothbrush and its positioning. In addition, the central drive system allowed for consistency and reproducibility of the protocol, eliminating human variables in speed and force used when brushing.

Consistent with previous findings [5], Fuji IX and EQUIA Forte HT released the highest fluoride levels, with all materials showing a decline over time, which is in accordance with other studies [29,30]. This decline varied by material, with significant differences in fluoride release levels (*p* < 0.001) and their rates of change (*p* < 0.001) across materials. Similarly, toothpaste type influenced fluoride release, with significant overall differences (*p* < 0.001) and varying rates of change over time (*p* < 0.001). Thus, both material composition and toothpaste affected fluoride release dynamics at different time points.

As fluoride release declines over time, limiting the material’s anti-cariogenic potential, fluoride recharging via oral hygiene products may be more critical than initial release [31,32,33]. Fuji IX and Fuji II LC, both modified glass ionomer cements, exhibited the ‘burst effect’, releasing high fluoride levels within the first 24 h, indicating enhanced uptake [34,35]. Although often considered beneficial for high-risk patients, this claim is debatable, as most fluoride remains bound within the cement [36,37]. In the current study, conventional GIC showed higher capacity for fluoride uptake, compared to RMGIC. However, no clear evidence explains why one material should release more ions than the other, as studies show similar fluoride release performance [38,39]. It comes as no surprise that fluoride release from these materials is also significantly boosted using NaF-based toothpastes, similar to EQUIA Forte HT, as glass hybrids developed from glass ionomer technology. To our best knowledge, EQUIA Forte HT (along with its predecessor EQUIA Forte) is currently the only glass hybrid material available on the market, with proven strong ion-releasing capacity [40,41]. This material, along with the standard glass fillers, contains much smaller fluoroaluminosilicate (FAS) glass fillers [42]. Smaller fluoride-containing fillers increase the surface area for dissolution, promoting fluoride diffusion into the cement matrix for subsequent release [43,44,45]. However, when coated, fluoride release decreases, due to coating protecting and preventing the material from degradation [46]. Cention Forte, a resin-containing alkasite material, showed improved fluoride uptake and release for most of the toothpastes tested. This consistency indicates that Cention Forte is more responsive to the fluoride content and formulations of the toothpastes used in this study. Fluoride release from Activa and Luminos UN, ion-releasing composite materials, were less affected by different toothpastes. Composites release fluoride through gradual salt dissolution, which is stable and minimally affected by toothpaste abrasivity or pH due to their dense, non-porous resin matrix. In contrast, glass ionomers, with their porous structure, release fluoride via ion exchange and surface degradation, making them more sensitive to toothpaste composition and physical properties [47,48]. Lower fluoride uptake by composite materials is in accordance with the findings of Gui et al. [49]. However, other studies noted that fluoride-containing composites are capable of creating fluoride complexes with the ions introduced from toothpastes [50,51]. These larger fluoride complexes face greater movement resistance, leading to prolonged retention within the resin matrix. This slower release suggests that resin composites may sustain fluoride release over an extended period, potentially enhancing their ability to prevent recurrent caries as the fluoride availability increases with time [52]. In contrast, Weidlich et al. [53] reported that composite resins lack fluoride uptake capacity altogether. Discrepancy in the results most likely stems from the fact that the mentioned study did not include any brushing protocol or fluoride-containing toothpastes; it merely relied on storing the samples in artificial saliva.

Toothpastes with higher sodium fluoride concentrations (Duraphat 2800 and Duraphat 5000) resulted in greater fluoride uptake. This is primarily because NaF is highly water-soluble, whereas SnF only partially ionizes [54]. However, SnF strongly binds to material components, providing long-term protection against acidic degradation [55]. In addition, it should be noted that previous research confirmed that Sn2F-based toothpastes outperform other combinations in the clinical setting for lesion remineralization, which this study did not aim to replicate [56,57].

The control group served as a baseline to measure natural fluoride release from each material. This setup isolated the effects of toothpaste and brushing, allowing for comparisons with experimental groups to identify any significant changes in fluoride release caused by these factors. Differences in release levels between control and experimental groups highlight the influence of toothpaste composition and mechanical brushing. In addition, the group treated with Parodontax toothpaste, containing no fluorides, also showed increased capacity for fluoride uptake. This suggests that toothbrushing alone could potentially lead to elevated fluoride release and uptake.

Evaluation of the effects of different toothpastes on fluoride uptake shows that those containing NaF show superior results to those with other active ingredients for the aforementioned reasons. However, Nicholson et al. [22] noted that the specific formulation of the fluoridating medium is relatively insignificant, as the strong affinity of the materials for fluoride is sufficient to counteract any interactions between fluoride ions and other components within these mixtures.

Ra is the most widely used roughness parameter, providing a straightforward arithmetic mean of surface deviations. It is easy to measure, interpret, and compare across studies. The results suggest that all included toothpastes have increased the surface roughness, with the biggest change generally observed for the group treated with Parodontax, which declaratively contains no fluorides. Previous studies show a positive correlation between surface roughness and fluoride release, and consequently, fluoride uptake, particularly in glass ionomer materials [58], which is most observed in glass ionomer materials. A rougher surface increases the available area and active sites for fluoride ion adherence, enhancing their incorporation into the material [59].

Similarly, all toothpastes reduced material surface microhardness, except in the coated EQUIA Forte HT group. This likely occurred because brushing removed softer superficial resin-based varnish layers, reducing thickness and increasing compactness. The abrasiveness of toothpastes is a well-known factor for the wear of material during toothbrushing protocols, which occurs mostly through micro cutting, microfracture, and repeated deformations [60]. In the current study, the lowest microhardness decrease was observed in the Luminous and Activa group, both composite materials, which, as a result, also showed the lowest potential for fluoride uptake. This further suggests the positive connection between moderate microhardness decrease and elevated fluoride uptake.

There are several factors that could possibly affect the results of the current study. Toothpastes vary in their abrasive particles, such as hydrated silica, calcium carbonate, or sodium bicarbonate, which can affect the material’s surface and potentially alter fluoride release. Abrasive formulations may increase surface roughness, which can affect ion exchange and fluoride release rates [4,61]. Furthermore, glass-ionomer cements typically release more fluoride due to their inherent chemical structure, which allows for greater ion exchange compared to resin-based composites [62]. The pH value of the toothpaste can also affect the outcomes of the study, as more acidic formulations could lead to higher ion exchange levels, as well as reduce the microhardness and increase surface roughness of the material. However, such toothpastes are usually intended for whitening purposes and were not included in this study. The duration and force of brushing, as well as the frequency of exposure to fluoride-containing media, can also influence the amount of fluoride released over time. The results of studies on fluoride release from dental materials can be influenced by the type of medium used in laboratory settings. For instance, many studies rely on artificial saliva, distilled water, or specific fluoride solutions to simulate the oral environment. However, these artificial mediums may not fully replicate the complex dynamics of the human mouth, which include variations in pH, the presence of food particles, and different types of oral bacteria. For example, fluoride release rates may differ when tested in acidic environments, which mimic conditions after consuming acidic drinks, compared to neutral pH environments like artificial saliva [63].

This study provides key insights into fluoride release dynamics in dental restorations, emphasizing material- and toothpaste-dependent differences. These findings have direct implications for dental practice, suggesting that a tailored approach to toothpaste selection, based on the specific restorative material, could optimize fluoride release and enhance caries prevention. The study also emphasizes the need for ongoing fluoride supplementation, given the observed decline in release over time, to maintain long-term oral health benefits.

While this study provides valuable initial data, several factors may influence the results and are the limitations of this study, including experimental protocols, bioactive ingredient concentrations in toothpastes, and environmental conditions. Notably, the four-day duration may be insufficient to fully assess long-term ion uptake. Long-term studies are needed to explore how fluoride release changes over months or years, beyond the four-day period studied here. Additionally, investigating the mechanisms underlying the interaction between specific materials and toothpastes could provide deeper insights into optimizing fluoride uptake and release.

## 5. Conclusions

The results of this study suggest that fluoride uptake, surface roughness, and microhardness changes in restorative materials after brushing were significantly influenced by both the material type and toothpaste composition. Fuji IX and EQUIA Forte HT showed the highest fluoride uptake, particularly when used with sodium fluoride toothpastes (Duraphat 5000/2800), while resin composites exhibited lower but more stable fluoride release. Brushing increased surface roughness in all materials, with non-fluoridated toothpaste (Parodontax) causing the most pronounced effect, likely due to its abrasive properties. Microhardness decreased across all groups except coated EQUIA Forte HT.

## Figures and Tables

**Table 1:** Restorative materials used in the study.

Commercial Name	Type of the Material	Composition	Manufacturer	Lot No.
Cention Forte	Alkasite (resin composite with reactive glass fillers)	Urethane Dimethacrylate (UDMA), Dicalcium Phosphate (DCP), Aromatic aliphatic-UDMA, Polyethylene Glycol 400 Dimethacrylate (PEG-400 DMA), Barium aluminium silicate glass,	Ivoclar Vivadent, Schaan, Liechtenstein	Z005MC
Luminos UN	Fluoride-containing nano hybrid composite	Bisphenol A Glycidyl Methacrylate/Triethylene Glycol Dimethacrylate (Bis-GMA/TEGDMA) resin, multifunctional filler (including micronized fluoroboroaluminosilicate glass)	UnoDent Ltd., Witham, United Kingdom	20250225
EQUIA Forte HT, without coat	Glass hybrid	Powder: fluoroaluminosilicate glass, polyacrylic acid, iron oxide Liquid: polybasic carboxylic acid, water	GC Corporation, Tokyo, Japan	230310B
EQUIA Forte HT, with coat	Glass hybrid, covered with resin-based coat	Coat: silica fillers, multifunctional monomers. Powder and liquid same as EQUIA Forte HT.	GC Corporation, Tokyo, Japan	230310B
ACTIVA-BioACTIVE-Restorative	Bioactive composite	Diurethane dimethacrylate (UDMA) and other methacrylate-based monomers, polyacrylic acid/maleic acid copolymer, silanated bioactive glass and calcium, silanated silica, sodium fluoride, aluminoflurosilicate ionomer glass, and water	Pulpodent, Watertown, MA, USA	160209
Fuji II LC	Resin-modified glass ionomer material	Fluroaluminisilicate glass/ Liquid distilled water, polyacrylic acid, Hydroxyethyl Methacrylate (HEMA), UDMA	GC Corporation, Tokyo, Japan	230105A
Fuji IX GP Extra (Fuji IX)	Conventional glass ionomer material	Liquid: Distilled water, Polyacrylic acid Powder: Fluoroaluminosilicate glass (FAS)	GC Corporation, Tokyo, Japan	240402A

**Table 2:** Toothpastes used in the study.

Toothpaste	Manufacturer	Declared Fluoride	Declared ppm F^−^
Duraphat 5000	Colgate-Palmolive, Mortagne, France	Sodium fluoride	5000
Duraphat 2800	Colgate-Palmolive, Mortagne, France	Sodium fluoride	2800
Colgate Total	Palmolive, NY, USA	Sodium fluoride	1450
Oral B Gum & Enamel	Procter & Gamble, Schwalbach TS, Germany	Stannous fluoride, Sodium fluoride	1100 350
Elmex Sensitive professional	GABA InternationalAG, Therwil, Switzerland	Sodium monofluorophosphate	1450
Parodontax Classic	Haleon Medical Devices, Dungarvan Co., Waterford, Ireland	No declared fluoride	/

## Data Availability

The raw data supporting the conclusions of this article will be made available by the authors on request.

## Abbreviations

The following abbreviations are used in this manuscript:

GICs	Glass Ionomer Cements
NaF	Sodium Fluoride
SnF2	Stannous Fluoride
SMFP	Sodium Monofluorophosphate
ppm	Parts Per Million
UDMA	Urethane Dimethacrylate
DCP	Dicalcium Phosphate
PEG-400 DMA	Polyethylene Glycol 400 Dimethacrylate
Bis-GMA	Bisphenol A Glycidyl Methacrylate
TEGDMA	Triethylene Glycol Dimethacrylate
HEMA	Hydroxyethyl Methacrylate
FAS	Fluoroaluminosilicate
RMGIC	Resin-Modified Glass Ionomer Cement
HV	Vickers Hardness
LSD	Least Significant Difference
SPSS	Statistical Package for the Social Sciences
